# Postdigital Research: Genealogies, Challenges, and Future Perspectives

**DOI:** 10.1007/s42438-022-00306-3

**Published:** 2022-03-28

**Authors:** Petar Jandrić, Alison MacKenzie, Jeremy Knox

**Affiliations:** 1 Zagreb University of Applied Sciences, Zagreb, Croatia; 2 University of Wolverhampton, Wolverhampton, UK; 3 Queen’s University, Belfast, UK; 4 University of Edinburgh, Edinburgh, UK

**Keywords:** Postdigital, Research, Philosophy, Epistemology, Methodology, Praxis, Digital, Analog, History

## What Binds Us Together?

Since the inception of *Postdigital Science and Education* journal and book series, postdigital scholarship has experienced rapid growth. However, the implications of postdigital research (methods) have remained unclear. What does it mean to conduct postdigital research? What are the main challenges and opportunities of postdigital research? And, probably most importantly, what are its prospects for the future?

This essay addresses the *Postdigital Science and Education* community’s pressing need to examine its own research praxes and asks: What binds us together in postdigital research, particularly in a methodological sense? The essay outlines genealogies, challenges, opportunities, and future perspectives of postdigital research. It offers our understanding of the main developments in the field and serves as a supplement to the call for papers for our forthcoming edited book on postdigital research.

## A Brief Genealogy

The earliest appearance of the postdigital idea is generally attributed to Nicholas Negroponte. In a seminal Wired article, Negroponte wrote that the digital is designed for the invisibility (and therefore banality) caused by its omnipresence in our daily lives. ‘Its literal form, the technology, is already beginning to be taken for granted, and its connotation will become tomorrow’s commercial and cultural compost for new ideas. Like air and drinking water, being digital will be noticed only by its absence, not its presence.’ (Negroponte 1998) Soon after Negroponte’s articulation of the inseparability of the digital and the analog, his idea acquired its own name and started its journey into the history of thought.

The word postdigital was first mentioned in Cascone essay ‘The Aesthetics of Failure’ (2000), and Pepperell and Punt book *The Postdigital Membrane: Imagination, Technology and Desire* (2000). Without knowledge of each other’s work, these authors developed the concept in the context of music (Cascone) and visual art (Pepperell and Punt) (more about early history of the concept can be found in Cascone and Jandrić 2021). In the following years, the concept of the postdigital occasionally appeared across wide arts-related fields such as music, visual arts, architecture, design, and so on.

In 2014 Christian Ulrik Andersen, Geoff Cox, and Georgios Papadopoulos made the first known attempt at systematization in a Special Issue titled ‘Post-digital Research’.^1^ In the introduction to the Special Issue, they take the concept of the postdigital ‘to be a serious concept that deserves our critical attention’ and

address the term itself, its genealogy and wider connotations, as well as its potential usefulness across different fields (including art, acoustics, aesthetic theory, political economy and philosophy). Given that the term comes from practice, [the Special Issue] also addresses how the postdigital potentially operates as a framework for practice-based research that relate to material and historical conditions. (Andersen et al. 2014: 5)

This Special Issue marks the ‘official’ entrance of postdigital research into mainstream academia; yet its focus and scope remained largely within artistic fields and practice-based research.

Two decades are a long time in artistic practice. In 2021 Cramer (in Cramer and Jandrić 2021: 977) wrote that, ‘in the meantime, the term postdigital has become rather useless in the arts, because it is constantly being conflated and confused with the too-similar-sounding and much better-known “Post-Internet”’. However, continues Cramer, the concept still offers a lot beyond its fields of origin:

The best possible contribution of the concept ‘postdigital’ in 2021 is, in my opinion, that it can help to complicate the terms ‘digital’ and ‘analog’, particularly in the humanities and social sciences. It could also be used for fields of technology that are literally postdigital, such as biocomputing and continuous variable quantum computing. (Cramer in Cramer and Jandrić 2021: 985)

In 2018 a similar line of thinking inspired the inception of the *Postdigital Science and Education* journal and book series (see Jandrić et al. 2018). Following the footsteps of many scholarly concepts which originated in the arts, the concept of the postdigital has now become an integral part of scholarly discourse. *Postdigital Science and Education* is arguably the main publishing ecosystem for postdigital research, though far from being the only one.^2^ Today, postdigital research centres,^3^ projects,^4^ and publications are growing in number^5^ and importance.^6^

## Against Definitions

The community has occasionally, but far from systemically, dealt with the history of the concept (Cormier et al. 2019); its relationships to other ‘schools of thought’ (Knox 2019; de Laat and Dohn 2019); terminology (Sinclair and Hayes 2019; Fawns 2019); methodology (Jandrić 2020a, 2020b, 2021; Jandrić and Knox 2021; Ryberg 2021; Macgilchrist 2021); and other elements typically required of a distinct ‘field’, ‘perspective’, ‘school of thought’, or ‘discipline’. We place these terms under quotation marks, as we do not think that they fully apply to postdigital thought. The question ‘What is postdigital?’ is simultaneously both useful and preposterous, both friend and foe.

The question is a friend because it helps us to focus our thoughts and ideas; it is a foe, because ‘[t]o define a field is necessarily to put boundaries around it, to determine which writings, conversations, people are “inside” and which are “outside”’ (Bayne in Networked Learning Editorial Collective et al. 2021: 333). This is why Jandrić and Ford argue that.

one day, probably, our postdigital condition will be condensed in concise encyclopaedia entries and routinely explained by undergraduates. One task is to ensure this does not happen, and that the postdigital remains—for as a long as it is productive—a concept that constantly resists any final definition. (Jandrić and Ford 2020)

This is also why the only thing the community has clearly defined is spelling. While the word postdigital can be found in various versions, such as postdigital, post digital, and so on, for pragmatic reasons, it was decided to maximize online searchability and accessibility by consistent use of the spelling ‘postdigital’. This decision is significant as a postdigital move itself: rendering the term ‘easy to type into search boxes’ as well as ‘simple to be indexed in databases’ is a demonstration of complicating the distinction between digital and analog. The term for this ‘school of thought’ was decided upon, at least in part, due to the functioning of digital infrastructure.

The concept of the postdigital deliberately defies any attempts at definition, yet its history and genealogy strongly determine its research practices. These practices range anywhere from arts-based research, through humanities and social sciences, to natural sciences and engineering. Some of this epistemic anarchy (Feyerabend 1970/1993) can be attributed to troubling distinctions and boundaries across disciplinary research approaches, which now attract a lot of attention in the journal (see Gibbs 2021; MacKenzie 2022; Green 2022). What are the other possible sources of the ‘Jack-of-all-trades’ nature of postdigital research? And how can we avoid the much less pleasant continuation of the adage, ‘and master of none’?

## The Curious Dance Between Epistemology and Methodology

Postdigital research can employ any research methodology, yet not all research is postdigital. Postdigital research may not even mention the word postdigital, and research articles using the word postdigital may misinterpret its philosophy. This makes tasks such as editing and reviewing messy; despite our wariness of definitions, this mess cannot be cleared up without some conceptual clarity. In the mission statement article for the journal and the book series, it was decided, therefore, to embrace this messiness as an inherent part of the postdigital condition. ‘The postdigital is hard to define; messy; unpredictable; digital and analog; technological and non-technological; biological and informational. The postdigital is both a rupture in our existing theories and their continuation.’ (Jandrić et al. 2018: 894).

Conscious of the messy nature of our postdigital condition, and wary of Bayne’s warning that even the best definitions will also determine what postdigital research is not (Bayne in Networked Learning Editorial Collective et al. 2021: 333), this article and our forthcoming edited book on postdigital research actively avoid defining, delineating, and canonizing postdigital research to any restrictive extent. In the language of epistemology, we despise ‘ownership’ of the concept characteristic of knowledge capitalism and situate our work firmly within the idea of knowledge as a common construct and a common good characteristic for knowledge socialism (Peters et al. 2020).

Our attitude does not imply epistemic relativism, at least not in the form of ‘anything goes’. But we insist that the concept of the postdigital must remain a common good, and we insist that the community of postdigital researchers needs to be open to everyone who does good work — including non-human agents. This is a community that is open to what constitutes knowledge, and the means by which to attest, authenticate, or audit the truthfulness of its creation. In this essay, therefore, our aim is to introduce, challenge, provoke, and start a wide postdigital dialogue (Jandrić et al. 2019) about the present and future of research in our postdigital age, while sustaining the elasticity and capaciousness of the concept.

## An Invitation to Dialogue

Though the aim is to avoid restrictions and canonization, and to retain the capaciousness of the concept, we nevertheless need to describe the postdigital in a meaningful and widely applicable way. We need, further, to head off challenges which could allege that in being as open as we claim to be to the construction and production of knowledge, there is no end to what could be asserted as ‘research’, and in the process lose credibility. As noted by contributors to a current discussion on postdigital transdisciplinarity (see Gibbs 2021; MacKenzie 2022; Green 2022), research within the disciplines can be hyperspecialised and compartmentalised, lacking flexibility in addressing urgent global problems. While we want to be careful that we don’t go down the same route, disciplines are important, as they rely on research processes that address rigour, validity, trustworthiness, and so on.

We have to take a stand on what we mean by quality, credible, trustworthy postdigital research. The very fact that we talk of ‘postdigital’ rather than postdigital sociology, postdigital politics, postdigital music, or postdigital philosophy, with their key and emerging thinkers, methods of enquiry, and favoured research designs, may help us; ‘postdigital’ is a guiding idea, rather than an absolute or definitively bounded concept. We are well aware that the concept of the postdigital was developed in response to certain socio-technological conditions of our space–time. As soon as these conditions change, postdigital research will inevitably transform or give way to more appropriate approaches and terms.

We accept impermanence and contextuality as an inherent part of knowledge development. We warmly welcome all kinds of criticism, including the harshest rebuttals, and we humbly embrace our ephemeral role in the history of human thought. Yet we are well aware that our tiny role arrives with a huge responsibility. Researchers of today and tomorrow need strong shoulders to stand on; it is our duty to scaffold the postdigital research of the present and bequeath our best to the future.

Following our belief that it takes a village to raise a child, we decided to frame our research in a widely open postdigital dialogue (Jandrić et al. 2019). All knowledge is co-constituted by the conditions of its making, and our efforts are shaped in the form of an edited book. This setup may result in a mashup of openings and closures, opportunities and restrictions, orientations to both the empirical and theoretico-philosophical, which we hope to critically interrogate along the way. We invite the postdigital community to join us in these efforts and examine the scope, importance, methodology, and significance of its present research praxes — while firmly keeping an eye on the future.

## Footnotes

1 See https://aprja.net//issue/view/8400. Accessed 10 February 2022.

2 See, for instance, The Palgrave Studies in Educational Media book series edited by Eckhardt Fuchs and Felicitas Macgilchrist. https://link.springer.com/series/15151. Accessed 10 February 2022.

3 Some of the most prominent centres in the humanities and social sciences include Coventry University's Centre for Postdigital Cultures (https://www.coventry.ac.uk/research/areas-of-research/postdigital-cultures/) and The Leibniz Science Campus—Postdigital Participation – Braunschweig (https://www.postdigitalparticipation.org/en/). Accessed 10 February 2022.

4 Alongside many projects in the humanities and social sciences, a good example in science and engineering is the European Training Network on Post Digital Computing – POSTDIGITAL (https://postdigital.astonphotonics.uk/). Accessed 10 February 2022.

5 In less than four years of publication, *Postdigital Science and Education* journal has published more than 300 articles. Founded in 2021, the Postdigital Science and Education book series now has five books and many more in the making. See https://www.springer.com/series/16439. Accessed 10 February 2022.

6 With 526,200 downloads in 2021, *Postdigital Science and Education* is positioned amongst the most read journals in education and education technology. The journal also has a high citation count, and the journal’s most cited article, ‘Online university teaching during and after the Covid-19 crisis: Refocusing teacher presence and learning activity’ (Rapanta et al. 2020), has amassed almost 200 000 downloads and 1000 citations in less than two years. See journal’s real-time Google citation metrics at https://scholar.google.com/citations?user=RyZRb5IAAAAJ&hl=en. Accessed 10 February 2022. *Postdigital Science and Education* articles regularly appear in prestigious lists such as the Dublin City University's National Institute for Digital Learning list of top 10 'good reads'. See https://nidl.blog/2022/01/10/good-reads-from-2021-our-nidl-top-10-journal-articles-part-3/. Accessed 10 February 2022. *Postdigital Science and Education* articles have been republished in several languages including Chinese, Croatian, German, Spanish, and Turkish. In 2022, its fourth year of publication, Postdigital Science and Eucation is listed by Scopus. 
